# Genetic adaptation of *Streptococcus mutans *during biofilm formation on different types of surfaces

**DOI:** 10.1186/1471-2180-10-51

**Published:** 2010-02-18

**Authors:** Moshe Shemesh, Avshalom Tam, Reuven Aharoni, Doron Steinberg

**Affiliations:** 1 Biofilm Research Laboratory, Institute of Dental Sciences, Faculty of Dental Medicine, Hebrew University-Hadassah POB 12272, Jerusalem 91120, Israel; 2 Current address: Department of Molecular and Cellular Biology, Harvard University, Cambridge, MA 02138, USA

## Abstract

**Background:**

Adhesion and successful colonization of bacteria onto solid surfaces play a key role in biofilm formation. The initial adhesion and the colonization of bacteria may differ between the various types of surfaces found in oral cavity. Therefore, it is conceivable that diverse biofilms are developed on those various surfaces. The aim of the study was to investigate the molecular modifications occurring during *in vitro *biofilm development of *Streptococcus mutans *UA159 on several different dental surfaces.

**Results:**

Growth analysis of the immobilized bacterial populations generated on the different surfaces shows that the bacteria constructed a more confluent and thick biofilms on a hydroxyapatite surface compared to the other tested surfaces. Using DNA-microarray technology we identified the differentially expressed genes of *S. mutans*, reflecting the physiological state of biofilms formed on the different biomaterials tested. Eight selected genes were further analyzed by real time RT-PCR. To further determine the impact of the tested material surfaces on the physiology of the bacteria, we tested the secretion of AI-2 signal by *S. mutans *embedded on those biofilms. Comparative transcriptome analyses indicated on changes in the *S. mutans *genome in biofilms formed onto different types of surfaces and enabled us to identify genes most differentially expressed on those surfaces. In addition, the levels of autoinducer-2 in biofilms from the various tested surfaces were different.

**Conclusions:**

Our results demonstrate that gene expression of *S. mutans *differs in biofilms formed on tested surfaces, which manifest the physiological state of bacteria influenced by the type of surface material they accumulate onto. Moreover, the stressful circumstances of adjustment to the surface may persist in the bacteria enhancing intercellular signaling and surface dependent biofilm formation.

## Background

Microbial adhesion onto surfaces and the subsequent formation of biofilms are critical concerns for many biomedical and dental applications. The initial adhesion and the successful colonization of bacteria onto solid surfaces play a key role in biofilm formation and the pathogenesis of infections related to biomaterials [1-4]. Many bacteria prefer to exist predominantly attached to surfaces in contact with liquids [5]. The advantages gained by the bacteria immobilized on surfaces are thought to include increased protection from the host's immune system, higher protection against antimicrobial agents, higher concentration of nutrients close to a surface, and easier inter cellular genetic and signal exchange [6].

The oral cavity is a unique environment, as different types of surfaces (hard, soft, natural and artificial) share the same ecological niche. In order to survive within this 'open growth system' and to resist shear forces, bacteria need to adhere either to soft or hard tissues [7,8]. Adhesion of oral bacteria to acquired enamel pellicle (AEP) leads to the development of the dental plaque biofilm. AEP is a-cellular film which results from selective adsorption of bacterial and host constituents such as salivary components. Among the artificial surfaces in the mouth one can find various types of restorative materials, which differ in chemical and physical properties. Although these surfaces occur in the same ecological niche, the attached biofilms are probably substantially different from one another, and each of these biofilms represents a unique micro-environment [9].

Potentially, each type of surface, such as implant, restorative material, or enamel, can be associated with the formation of different type of a biofilm. Clearly, controlling the initial adhesion into a biofilm depends mainly on the surface properties. While several dental materials promote selective adherence during early dental biofilm formation [10,11], other modified biomaterials may provide resistance to bacterial adhesion and biofilm formation [12,13]. Therefore, it is expected that diverse biofilms are developed on various surfaces. Previous studies have demonstrated that streptococci, including mutans streptococci, are the predominant colonizing microorganisms of oral surfaces. *S. mutans *is considered to be a most important etiological agent of diseases associated with dental caries. On teeth, it is one of the species which form biofilm causing dissolution of enamel by acid end-products resulting from carbohydrate metabolism [14-16].

In nature, acclimation of bacteria to any type of biofilm environment is probably associated with a change in gene expression [17-19]. However, in contrast to other areas, less is known about the gene expression of bacteria immobilized on different dental surfaces. It is compelling that adaptation of oral bacteria to the different types of dental surfaces may also be associated with different patterns of gene expression, especially those genes associated with biofilm regulation, formation and bacterial physiology. The aim of this study was to identify transcriptional modifications that accompany the formation of *in vitro *biofilms by *S. mutans *on a variety of dental surfaces.

## Methods

### The tested surfaces

Dental restorative material - composite Filtek Z250 (60% zirconia/silica, average particle size 0.01-3.5 microns; BIS-GMA, UDMA and BIS-EMA resins (3 M Dental Products, St Paul, MN, USA)).

Ti disks tested in this study were Ti alloy (TiAl_(6)_V_(4)_) disks (6 mm diameter) with machined type of surface modifications manufactured by Alpha-Bio implant company (Petach Tikva, Israel).

Hydroxyapatite (HA) tablets were prepared by the following procedure: 340 mg of HA beads (Bio-Rad Laboratories, Hercules, CA, USA) of particle size diameter 80 μm, surface area 40 m^2^/g, were pressed at a pressure of 8 tons for 20 sec by a single-punch machine (Erweka, Frankfurt, Germany). The punch diameter was 1.2 cm. Before every preparation of tablets the punch (all the surface and inside) was cleaned with ethanol (70%) and stearic acid (5%).

Following the sterilization the Ti, HA, and the composite materials were placed into the 20-mm diameter and 15-mm deep polystyrene multidishes (NUNCLON-143982, Roskilde, Denmark); consequently, the polystyrene multidishes were used as a non-dental reference surface.

### Bacterial strains and culture conditions

*S. mutans *UA159, a serotype c strain, was obtained from Robert Burne (University of Florida, Gainesville). The planktonic *S. mutans *UA159 was grown in Brain Heart Infusion Broth (BHI, Difco Labs, Detroit, USA) at 37°C in 95% air/5% CO_2 _(v/v) in polystyrene tubes for 24 h. For biofilm generation, *S. mutans *culture was seeded in 20-mm diameter, 15-mm deep sterile polystyrene multidishes (NUNCLON-143982, Roskilde, Denmark), and cultivated with fresh BHI medium at 37°C in 95% air/5% CO_2 _(v/v) for 18 h. For generation of the biofilm on different surfaces, we placed the Ti, HA, or the composite into the polystyrene multidishes. Each experiment was performed in three independent biological repetitions in duplicates.

### Analysis of biofilm construction

The 18 h grown biofilms developed on the different surfaces were analyzed for depth and bacterial vitality using a confocal laser scanning microscope (CLSM). The biofilm was stained with LIVE/DEAD *Bac*Light fluorescent dye (Molecular Probes, OR) (1:100) for 10 min. Fluorescence emission of the PBS washed samples was measured using a CLSM (Zeiss LSM 510, Carl Zeiss Microscopy, Jena, Germany). In each experiment, exciting laser intensity, background level, contrast and electronic zoom size were maintained at the same level. At least three random fields were analyzed in each experiment. A series of optical cross-sectional images was acquired at 6.9- μm depth intervals from the surface through the vertical axis of the specimen, using a computer-controlled motor drive. 3-D confocal images were reconstituted and processed for display using Adobe Photoshop ver. 7.0 software (Shemesh *et al*., 2007).

### RNA extraction

Extraction of total RNA from *S. mutans *cells was performed as described previously [20]. In brief, biofilm-grown cells were suspended in TRI Reagent (Sigma-Aldrich, St. Louis, MO, USA) and dislodged by scraping into a 2-ml microcentrifuge tube containing 0.4 ml 1-mm-diameter glass beads (Sigma-Aldrich). The cells were disrupted with the aid of a Fast Prep Cell Disrupter (Bio 101, Savant Instruments, Inc., NY, USA), centrifuged and the RNA containing supernatant was supplemented with 1-Bromo-3-Chloropropane (BCP) (Molecular Research Center, Cincinnati, OH, USA). The upper aqueous phase was precipitated with isopropanol. After centrifugation, the resulting RNA pellet was washed with ethanol and resuspended in diethyl pyrocarbonate (DEPC)-treated water.

Because of the sensitivity of the PCR, residual contaminatingDNA was eliminated by incubation of the sample with RNase-free DNase (Promega, Madison, WI, USA)_. _The DNase was then inactivated by incubation at 65°C for 10 min, and the RNA was precipitated with ethanol and suspended in diethyl pyrocarbonate (DEPC)-treated water. The RNA concentration was determined spectrophotometricallyusing the Nanodrop Instrument (ND-1000, Nanodrop Technologies, Wilmington, DE, USA). The integrity of the RNA was examined by agarose-gel electrophoresis (data not shown).

### Microarrays design, cDNA labeling and hybridization

Figure S1 shows schematically the construction of DNA-microarray experiments for gene expression studies of biofilm on representative surfaces. The arrays consisted of 1948 70-mer oligonucleotides representing 1960 open reading frames (ORF) of *S. mutans *UA159 and additional control sequences. The probe labeling, hybridization and array data normalization were carried out as previously described [21]. In brief, cDNA was generated with random primers from total RNA and labeled indirectly with cy3 or cy5 dye. Hybridizations were performed against the samples from the polystyrene and composite surfaces in a reference design manner (Additional file 1, Figure S1). Slides were scanned using a Genepix 4000B scanner (Axon Ltd). Fluorescence intensities were quantitatively analyzed using GenePix Pro 4.1 software (Axon). The result files (gpr) produced by GenePix were analyzed utilizing the LIMMA [22] software package, available from the CRAN site http://www.r-project.org. Spots flagged as not found or absent in GenePix were removed by filtering. Another filter was applied for saturated spots. After filtering, the data within the same slide were normalized using global loess normalization with the default smoothing span of 0.3 [23]. To identify differentially expressed genes, a parametric empirical Bayesian approach implemented in LIMMA was used [24]. According to this approach, data from all the genes in a replicate set of experiments are combined into estimates of parameters of a priori distribution. These parameter estimates are then combined at the gene level with means and standard deviations to form a statistic B that is a Bayes log posterior odds [24]. B can then be used to determine whether differential expression has occurred. A moderated *t *test was performed in parallel, with the use of a false discovery rate [25] correction for multiple testing. TIGR arrays included four replicates for each gene. Instead of taking the average of replicate spots, we used the duplicate correlation function [26] available in LIMMA to acquire an approximation of gene-by-gene variance. This method greatly improves the precision with which the gene-wise variances are estimated and thereby maximizes inference methods designed to identify differentially expressed genes. A *P *value < 0.05 confidence level was used to pinpoint significantly differentiated genes. Genes had to have an *A*-value (A = log_2 _[Cy3 × Cy5]/2), the average expression level for the gene across all arrays and channels) of more than 8.5, thus omitting genes with an average intensity in both channels of less than 256.

### Reverse transcription and real-time quantitative PCR

The quantitative SYBR green PCR assays employing an ABI-Prism 7000 Light Cycler System (Applied Biosystems, Foster City, CA, USA) was performed as described previously [14]. The corresponding oligonucleotide primers were designed using the algorithms provided by Primer Express (Applied Biosystems) for uniformity in size (≈ 90 bp) and melting temperature. For each set of primers, a standard amplification curve was plotted (critical threshold cycle against log of concentration) and only those with slope ≈ -3 were considered reliable primers. The expression levels of all the tested genes for real-time RT-PCR were normalized using the 16S rRNA gene of *S. mutans *(Acc. No. X58303) as an internal standard (Additional file 2, Table S1). Each assay was performed with at least two independent RNA samples in duplicate.

### Autoinducer-2 (AI-2) assay

It has been suggested [27,28] that AI-2 signaling may play an important role in the biofilm formation of *S. mutans*. It is conceivable that, the challenge of stressful condition during the transition to a new surface may alter the quorum sensing (QS) process in the bacteria. Consequently, we tested the secretion of AI-2 signal molecule by *S. mutans *immobilized in biofilms formed on the different surfaces to determine the impact of the tested material surfaces on the physiology of the attached bacteria. The AI-2 luminescence reporter assay was performed [29] to detect AI-2 secretion levels, in cell-free conditioned medium of *S. mutans *biofilms formed on the four tested surfaces. At the end of the biofilm incubation period, a supernatant fluid was collected and filtered through a 0.22 μm-pore size filter (Millipore). The cell-free conditioned medium was either used immediately or stored at -20°C. To determine the amount of AI-2, an overnight culture of *Vibrio harveyi *MM77, a mutant strain which does not produce either AI-1 nor AI-2, was diluted 1:5,000 in a mixture of 90% (v/v) fresh AB medium and 10% (v/v) conditioned medium to a total volume of 200 μl per well. The negative control contained bacteria in fresh AB medium alone and the positive control contained bacteria, fresh AB medium and 10% v/v spent medium containing AI-2 of *V. harveyi *BB152 (AI-1^-^, AI-2^+^). Readings were performed in triplicate in white 96-well plates with an optic bottom (NUNC) in a GENios reader (TECAN) at 30°C. Luminescence measurements were recorded every 30 min in parallel with optical density absorbance (*A*_595_) readings. The value of each reading (biofilm on various materials) was divided by the absorbance values to normalize the luminescence value of each sample to its cell density and to avoid dissimilarities caused by differences in growth rates. Fold induction above the non-specific luminescence background of the negative control was determined at the end of bacterial growth after approximately 15 hrs of growth. Fold induction in luminescence of each sample was normalized by the value of total fluorescence of live bacteria within the relevant biofilm as detected by CLSM.

## Results

**Figure 1:**
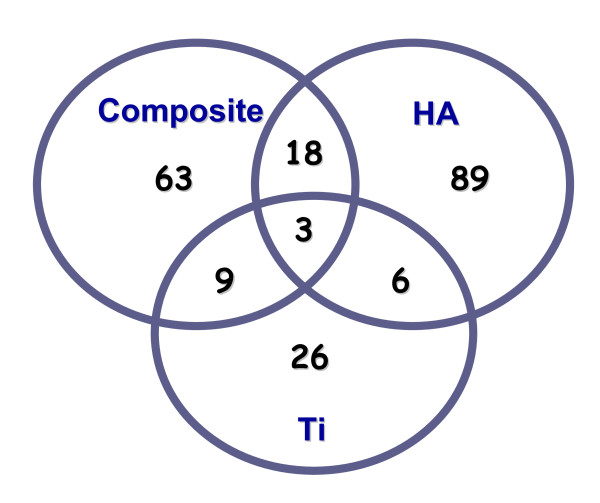
**Differentially expressed genes in biofilms formed on different surfaces**. Alignments of differentially expressed genes (*P *< 0.05) of *S. mutans *biofilms formed on hydroxyapatite, titanuim and composite (vs. polystyrene surfaces), showing the number of overlapping genes between the biofilms on different surfaces. Gene annotations are based on the genome information of *S. mutans *provided by TIGR.

**Figure 2:**
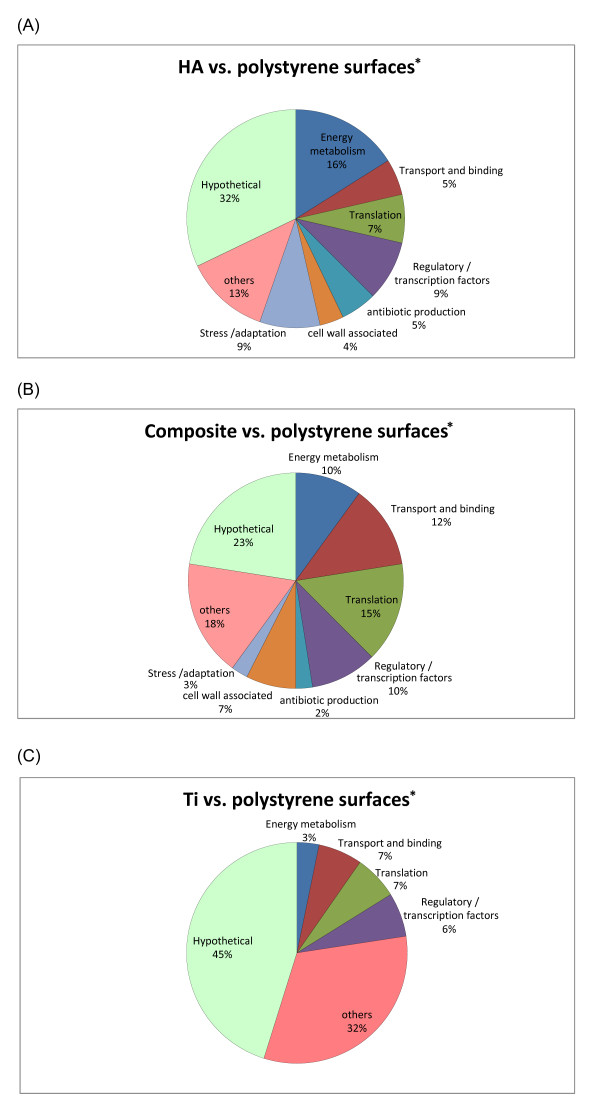
**functional categories of most differentially expressed genes**. Most significant (B* > 0) differentially expressed genes of *S. mutans*, grouped in functional categories, in biofilms formed on hydroxyapatite (A), titanium (B) and composite (C) vs. polystyrene surfaces. Gene annotations are based on information provided by TIGR. *Bayesian test value, i.e. the probability for a gene to be really differentially expressed.

**Figure 3:**
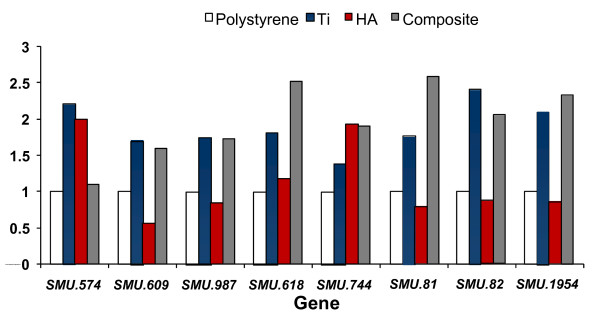
**Expression of selected genes analyzed by RT-PCR**. Comparison of RT-PCR expression values for selected genes of *S. mutans*, grown on different surfaces. SMU.81, SMU.82 (*dnaK*) and SMU.1954 (*groEL*) are stress-related genes; SMU.574c, SMU.609, and SMU.987 are associated with cell wall proteins. SMU.744 codes for FtsY, while SMU.618 codes for a hypothetical protein. The data are expressed as the means of at least two biologically independent experiments.

Using DNA-microarray technology we identified the differentially expressed genes of *S. mutans *(Figure 1), reflecting the physiological state of biofilms formed on the different biomaterials tested. An empirical Bayesian method (B-test) was applied to test for differential expression in biofilms on various surfaces. Analysis of the microarray images revealed a total of 116, 93 and 44 genes on HA, composite, and Ti, respectively, in comparison with polystyrene surfaces, were differentially transcribed at a confidence level of *P *< 0.05 (Additional file 2, Tables S2-S4). For simplicity, the mostly differentially expressed genes were grouped into functional categories (Figure 2), (i.e., fulfilling the criteria B > 0 by B-test and more than 1.5-fold change), in biofilms formed on hydroxyapatite, titanium and composite vs. polystyrene surfaces. Eight selected genes were further analyzed by real time RT-PCR (Figure 3). Criteria for gene selection were either highly up-regulated or highly down-regulated genes, associated with virulence, and of known function rather than hypothetical genes. Among the most regulated ones were genes associated with stressful environmental conditions andsynthesis of molecular chaperones, in addition to cell wall associated proteins and adhesion-promoting genes. The real-time RT-PCR analysis confirmed only partially the expression ratios determined by microarray technique.

**Figure 4:**
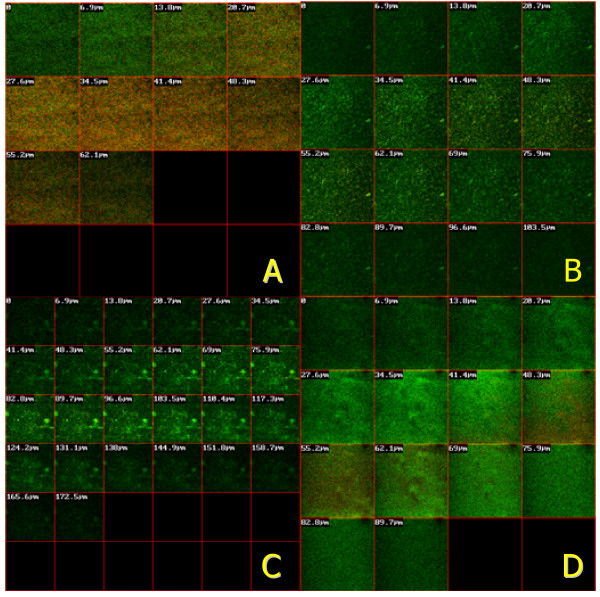
**Biofilms of *S. mutans *formed on different surfaces**. Biofilms of *S. mutans *UA159 were grown on different surfaces in BHI, stained with LIVE/DEAD *Bac*Light fluorescent dye and analyzed with CLSM. The panels show cross-section images of biofilms from polystyrene (A), Ti (B), HA (C) and composite (D) materials. Dead cells were stained red, and live cells were stained green.

To evaluate the physiological state of the immobilized bacterial populations generated on the different tested surfaces, the biofilms were characterized by using CLSM. Biofilm depth analysis showed that the bacteria were able to construct more confluent and profound biofilms on HA surface compared to other tested surfaces (Figure 4). According to the CLSM images, relatively little biofilm growth of about 62-micron depth was observed on the polystyrene surface (Figure 4c), whereas the biofilm formed on the HA surface was notably deeper, up to 173-micron depth (Figure 4b). Moreover, the vitality of the bacteria grown on the HA surface was much greater than those cultured on the polystyrene surface (Figure 4).

**Figure 5:**
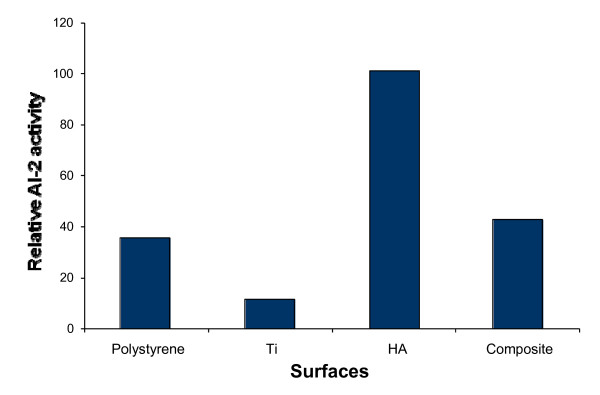
**AI-2 signal secretion by *S. mutans *biofilms on different surfaces**. Biofilms were grown on each material and the resulting conditioned media were exposed to *V. harveyi *MM77 for AI-2 bioassay. Fold induction in luminescence of each sample was calculated above background luminescence of the negative control (sample without addition of any conditioned medium) and was normalized by the value of total fluorescence of live bacteria within the relevant biofilm detected by CLSM.

To further determine the impact of the tested material surfaces on the physiology of the bacteria, we tested the secretion of AI-2 signal by *S. mutans *biofilms. As AI-2 reporter strain we used *V. harveyi *MM77, which does not produce endogenous AI-1 or AI-2. Thus, any increase of its luminescence above background level is due to exogenous AI present in the growth medium. The highest effect on the luminescence of the reporter strain was of the conditioned medium taken from biofilms grown on HA with normalized fold induction of ~100 per 10 million cells. Conditioned media from biofilms grown on composite and polystyrene had a similar effect on the luminescence resulting in normalized fold induction of ~40. The lowest effect on the reporter strain was of the conditioned medium taken from biofilm grown on titanium with normalized fold induction of only ~10 (Figure 5).

## Discussion

Mechanisms governing biofilm formation have generated considerable interest in the general biofilm field and also in dental-related biofilms [30-35]. Oral biofilms vary in both structure and function but share general characteristics. In order to persist within the oral ecosystem, the bacteria need to adhere to either soft or hard tissues and to overcome local shear forces. Although it is well documented that saliva constituents coat biological surfaces in the oral cavity, the principal aim of this study was to examine a genetic adaptation of bacteria upon immobilization on non-biological surfaces. Our results indicate that bacteria can sense their non-biological substrate and express different genes accordingly, probably as part of the adjustment to a new micro-environment. It is likely that the stressful situation conducts the bacteria to enhance the factors of successful adjustment to certain surface by activation of expression of certain combination of genes. This could explain the fact that bacteria are able to adjust to any surface by manipulating their gene expression pattern. Differences in formed biofilm depths and viabilities among the different materials might be due to their surface properties. Therefore, it is reasonable to assume that bacterial profile of gene expression differs on various surfaces, allowing the species to adapt to the different types of micro-environment.

Most recent global transcription and proteomic profiling has revealed several aspects of the physiological adaptations that *S. mutans *undergoes following attachment to and growth on surfaces [21,36-38]. Nevertheless, only a few comprehensive studies have compared the influence of surface materials on the gene expression of immobilized bacteria adhering to different dental biomaterials. It is conceivable that the chemistry of the surface on which the biofilm is formed would affect the properties of the biofilm. Recent gene expression profiling showed marked differences in gene responses of bone cells on smooth and rough titanium surfaces [39]. Additional studies demonstrated that the biodegradation of composite resins differentially impacts the growth and gene expression of *S. mutans *[40]. In addition, biomaterial surface chemistry affected biofilm formation, and polyethylene oxide significantly inhibited *S. epidermidis *biofilm formation *in vitro *[41]. In the current study, we have shown that gene expression differs in *S. mutans *biofilms formed on different surfaces, therefore likely changing the physiology and virulence of the immobilized bacteria. Our CLSM biofilm depth analysis shows that the bacteria were able to construct more confluent and thick biofilms on a hydroxyapatite surface compared to the other surfaces tested.

AI-2 is a furanone borate diester that is synthesized in many bacteria by the LuxS protein and detected in *Vibrio harveyi *by a periplasmic protein called LuxP. It was proposed to function as a universal quorum-sensing signal for interaction between different bacterial species [42]. It has been previously shown that the AI-2 level decreased in chemostat-grown *E. coli *cultures exposed to different stresses [43]. In addition, QS is likely involved in stress gene regulation in *Porphyromonas gingivalis *[44]. The consequences of these data may provide the potential link between the type of surface, QS and stress regulation in biofilm-grown bacteria. This might suggest that the attachment of bacteria to a particular surface may have altered the level of AI-2 signaling in the generated biofilm to overcome stressful conditions. Consistent with this hypothesis is that the levels of AI-2 in biofilms from various tested surfaces were found to be different (Figure 5). The stressful situation during the transition to a new surface apparently induces the bacteria to enhance the QS process to overcome the challenge by activating stress-related as well as biofilm-associated genes at the same time. Although small peptides termed competence stimulating peptides (CSP) are the main QS signaling molecules in *S. mutans *[45], It was shown that AI-2produced by *S. mutans *play a role in biofilm formation [27] and analogues of the AI-2 may affect biofilm formation of *S. mutans *[46]. Moreover, secretion of AI-2 of *S. mutans *is related to protein synthesis, stress, and cell division [28]. Accordingly, production of different amounts of AI-2 by *S. mutans *on the different surfaces could contribute to adaptation of the immobilized bacteria and their acclimation to the new micro-environment. The highest level of AI-2 was detected in the conditioned medium taken from biofilms grown on HA. This result is in consistence with the biofilm depth analysis showing that the bacteria were able to construct more confluent and profound biofilms on HA surface. However, the lowest amount of AI-2 was found in Ti biofilms, while bacteria still formed relatively confluent biofilm on this substrate. The differences between the AI-2 levels and biofilm thickness could be explained by alternative mechanisms of biofilm development which enable the bacteria to bypath AI-2 requirement to form confluent biofilm. It is apparent that AI-2, especially in gram positive bacteria, is not solely responsible for biofilm control and it may have other physiological effects on the immobilized bacteria.

The use of the array-based approach enabled us to study the complex interplay of the entire *S. mutans *genome simultaneously. We examined the pattern of gene expression as a reflection of the bacteria's physiological state influenced by biofilm formation on several representative types of dental materials. Differences in expression of the various genes provide an indication as to their function in biofilm formation, and may help to understand the different physiological pathways associated with this process. A substantial number of differentially expressed genes, such as SMU.574c, SMU.609, and SMU.987, are associated with cell wall proteins. SMU.987 encodes a cell wall-associated protein precursor WapA, a major surface protein [47], which modulates adherence and biofilm formation in *S. mutans*. Previous studies demonstrated that levels of *wapA *in *S. mutans *were significantly increased in the biofilm phase [48], whereas inactivation of *wapA *resulted in a reduction in cell aggregation and adhesion to smooth surfaces [49]. The *wapA *mutants have reduced cell chain length, a less sticky cell surface, and unstructured biofilm architecture compared to the wild-type [50]. The differential expression of those genes coding for cell wall associated proteins indicates their role in activation of initial biofilm formation and adjustment of the bacteria to various surfaces.

Additional differentially expressed gene SMU.618 which was found to be most significantly upregulated in biofilm formed on composite is annotated as hypothetical protein with unknown function. SMU.744, encoding the membrane-associated receptor protein FtsY, the third universally conserved element of the signal recognition particle (SRP) translocation pathway [51], was also found among the differentially expressed genes. SRP was first identified in mammalian cells, and later in bacteria, and it was further shown that components of the SRP pathway are universally conserved in all three domains of life [52]. The SRP pathway delivers membrane and secretory proteins to the cytoplasmic membrane or endoplasmic reticulum [53]. *S. mutans *remained viable but physiologically impaired and sensitive to environmental stress when *ftsY *and other genes of the SRP elements were inactivated [51]. The high regulation of FtsY in biofilms grown on different types of surface indicates that the SRP system is crucial for bacterial survival in the transition of bacteria from polystyrene to the other surfaces tested.

Our microarray data also show that stress-related genes, including SMU.81, SMU.82 (*dnaK*) and SMU.1954 (*groEL*), were differentially regulated within biofilms of *S. mutans *formed on the surfaces. It is known that these genes are intimately involved in the clearance of misfolded aggregates and premature polypeptides produced during stress. This result indicates that there is a firm correlation between the transition of bacteria from one type of surface to another and the stress response. One possible explanation of these differences could be because of the environmental stress encountered by the biofilm bacteria during the transition to dental surfaces rather than to the polystyrene. The challenge of stressful situations during the transition and adjustment to a new surface induces the bacteria to switch on surface dependent gene expression for successful adjustment to certain surface.

Interestingly, a minority of the differentially expressed genes showed more than 2.5-fold change between the different surfaces. However, even small changes in mRNA levels could have the biological potential to affect bacterial metabolism and physiology. Relatively small changes in the level ofexpression of one gene can be amplified through regulatory networks. and result in significant phenotypic alteration [54]It is noticeable that biofilm formation on different surfaces does not radically alter the transcriptome. However, closer assessment reveals that these changes in gene expression have the potential to profoundly affect cellular physiology, which adapts the bacteria in the biofilm formed on various surfaces. It should be remarked also that real-time RT-PCR results did not fully agree with the microarray data for selected genes. The most prominent differences between the array and RT-PCR approaches are probably due to the inherent technical variability of the microarray technique. Another reason for the residual variation between the two techniques could be associated with the incorporation of labeling compounds only for the microarray technique and the intrinsic dependence on the enzyme used for labeling [55].

By evaluating gene expression patterns in *S. mutans *following immobilization on different surfaces, we demonstrated that biofilm development is accompanied by significant transcriptional changes (Tables S1-3). However, the existence of a surface-dependent universal biofilm gene-expression pattern is still questionable. Nonetheless, our results suggest that genes associated with stressful environmental conditions and the synthesis of molecular chaperones, as well as cell wall-associated proteins and adhesion-promoting genes, seem to be responsible for biofilm generation on different surfaces. Biofilm formation as a complex developmental process is characterized by intricate interplay of gene expression pattern; hence, the bacteria have very sophisticated ways to be better adjusted to particular surface by manipulating their gene expression pattern. We have tested only representatives of dental surfaces natural (HA), implant (Ti) and restorative material (composite), it is conceivable that biofilm formation accompanied by gene and signal changes would occur also on other types of dental surfaces.

## Conclusions

Transcriptional profiling revealed broadly based changes in the patterns of gene expression during biofilm development of *S. mutans *on different solid surfaces, which manifest the physiological state of bacteria influenced by the type of attachment substance. Moreover, the stressful circumstances of adjustment to a particular surface may stimulate the bacteria to enhance intercellular signaling and surface dependent biofilm formation.

## Abbreviations

AHL: acyl homoserine lactone; AI-2: autoinducer-2; BHI: brain heart infusion; CLSM: confocal laser scanning microscope; EPS: extracellular polysaccharides; QS: quorum sensing; TIGR: The Institute for Genomic Research; HA: Hydroxyapatite; Ti: Titanium disks.

## Authors' contributions

MS planned and carried out the experiments, performed DNA-microarrays and real time RT-PCR analyses and wrote the original manuscript. AT assisted in biofilms generation, RNA extraction, RT-PCR and CLSM experiments. RA helped in set up and performing the AI-2 assay experiments. DS conceived the study and oversaw its execution; he also revised the manuscript critically for important intellectual content. MS and DS integrated all of the data throughout the study and crafted the final manuscript. All authors read and approved the final manuscript.

## Supplementary Material

Additional file 1 **Figure S1**. Schematic diagram showing construction of DNA-microarray experiments for gene expression studies of biofilms on various surfaces. Click here for file

Additional file 2 **Table S1**. Nucleotide sequences of primers for genes whose expression was compared. **Table S2**. The differentially expressed (*P *< 0.05) genes of *S. mutans *biofilms on HA vs. polystyrene surfaces. **Table S3**. The differentially expressed (*P *< 0.05) genes of *S. mutans *biofilms on composite vs. polystyrene surfaces. **Table S4**. The differentially expressed (*P *< 0.05) genes of *S. mutans *biofilm on Ti vs. polystyrene surfaces. Click here for file

## References

[B1] Gristina AG Biomaterial-centered infection: microbial adhesion versus tissue integration Science 1987 237 4822 1588 1595 10.1126/science.3629258 3629258

[B2] Palmer RJ Jr Gordon SM Cisar JO Kolenbrander PE Coaggregation-mediated interactions of streptococci and actinomyces detected in initial human dental plaque J Bacteriol 2003 185 11 3400 3409 10.1128/JB.185.11.3400-3409.2003 12754239 PMC155391

[B3] Gristina AG Hobgood CD Webb LX Myrvik QN Adhesive colonization of biomaterials and antibiotic resistance Biomaterials 1987 8 6 423 426 10.1016/0142-9612(87)90077-9 3427140

[B4] Hall-Stoodley L Costerton JW Stoodley P Bacterial biofilms: from the natural environment to infectious diseases Nat Rev Microbiol 2004 2 2 95 108 10.1038/nrmicro821 15040259

[B5] Palmer J Flint S Brooks J Bacterial cell attachment, the beginning of a biofilm J Ind Microbiol Biotechnol 2007 34 9 577 588 10.1007/s10295-007-0234-4 17619090

[B6] Donlan RM Biofilm formation: a clinically relevant microbiological process Clin Infect Dis 2001 33 8 1387 1392 10.1086/322972 11565080

[B7] Kolenbrander PE Oral microbial communities: biofilms, interactions, and genetic systems Annu Rev Microbiol 2000 54 413 437 10.1146/annurev.micro.54.1.413 11018133

[B8] Teughels W Van Assche N Sliepen I Quirynen M Effect of material characteristics and/or surface topography on biofilm development Clin Oral Implants Res 2006 17 Suppl 2 68 81 10.1111/j.1600-0501.2006.01353.x 16968383

[B9] Marsh PD Dental plaque: biological significance of a biofilm and community life-style J Clin Periodontol 2005 32 Suppl 6 7 15 10.1111/j.1600-051X.2005.00790.x 16128825

[B10] Rasperini G Maglione M Cocconcelli P Simion M *In vivo *early plaque formation on pure titanium and ceramic abutments: a comparative microbiological and SEM analysis Clin Oral Implants Res 1998 9 6 357 364 11429937 10.1034/j.1600-0501.1996.090601.x

[B11] Grossner-Schreiber B Griepentrog M Haustein I Muller WD Lange KP Briedigkeit H Göbel UB Plaque formation on surface modified dental implants. An in vitro study Clin Oral Implants Res 2001 12 6 543 551 10.1034/j.1600-0501.2001.120601.x 11737097

[B12] Cheng G Zhang Z Chen S Bryers JD Jiang S Inhibition of bacterial adhesion and biofilm formation on zwitterionic surfaces Biomaterials 2007 28 29 4192 4199 10.1016/j.biomaterials.2007.05.041 17604099 PMC5463736

[B13] Beyth N Houri-Haddad Y Baraness-Hadar L Yudovin-Farber I Domb AJ Weiss EI Surface antimicrobial activity and biocompatibility of incorporated polyethylenimine nanoparticles Biomaterials 2008 29 31 4157 4163 10.1016/j.biomaterials.2008.07.003 18678404

[B14] Shemesh M Tam A Feldman M Steinberg D Differential expression profiles of *Streptococcus mutans ftf*, *gtf *and *vicR *genes in the presence of dietary carbohydrates at early and late exponential growth phases Carbohydr Res 2006 341 12 2090 2097 10.1016/j.carres.2006.05.010 16764842

[B15] Marsh PD Dental plaque as a microbial biofilm Caries Res 2004 38 3 204 211 10.1159/000077756 15153690

[B16] Selwitz RH Ismail AI Pitts NB Dental caries Lancet 2007 369 9555 51 59 10.1016/S0140-6736(07)60031-2 17208642

[B17] Whiteley M Bangera MG Bumgarner RE Parsek MR Teitzel GM Lory S Greenberg EP Gene expression in Pseudomonas aeruginosa biofilms Nature 2001 413 6858 860 864 10.1038/35101627 11677611

[B18] Lamont RJ Bryers JD Biofilm-induced gene expression and gene transfer Methods Enzymol 2001 336 84 94 full_text 11398422 10.1016/s0076-6879(01)36581-3

[B19] Becker P Hufnagle W Peters G Herrmann M Detection of differential gene expression in biofilm-forming versus planktonic populations of *Staphylococcus aureus *using micro-representational-difference analysis Appl Environ Microbiol 2001 67 7 2958 2965 10.1128/AEM.67.7.2958-2965.2001 11425708 PMC92967

[B20] Shemesh M Tam A Steinberg D Expression of biofilm-associated genes of Streptococcus mutans in response to glucose and sucrose J Med Microbiol 2007 56 Pt 11 1528 1535 10.1099/jmm.0.47146-0 17965356

[B21] Shemesh M Tam A Steinberg D Differential gene expression profiling of Streptococcus mutans cultured under biofilm and planktonic conditions Microbiology 2007 153 Pt 5 1307 1317 10.1099/mic.0.2006/002030-0 17464045

[B22] Smyth GK Linear models and empirical Bayes methods for assessing differential expression in microarray experiments Stat Appl Genet Mol Biol 2004 3 1 Article 3 10.2202/1544-6115.1027 16646809

[B23] Smyth GK Speed T Normalization of cDNA microarray data Methods 2003 31 4 265 273 10.1016/S1046-2023(03)00155-5 14597310

[B24] Lonnstedt I Speed T Replicated microarray data Statistica Sinica 2002 12 1 31 46

[B25] Reiner A Yekutieli D Benjamini Y Identifying differentially expressed genes using false discovery rate controlling procedures Bioinformatics 2003 19 3 368 375 10.1093/bioinformatics/btf877 12584122

[B26] Smyth GK Michaud J Scott HS Use of within-array replicate spots for assessing differential expression in microarray experiments Bioinformatics 2005 21 9 2067 2075 10.1093/bioinformatics/bti270 15657102

[B27] Merritt J Qi F Goodman SD Anderson MH Shi W Mutation of luxS Affects Biofilm Formation in Streptococcus mutans Infect Immun 2003 71 4 1972 1979 10.1128/IAI.71.4.1972-1979.2003 12654815 PMC152054

[B28] Sztajer H Lemme A Vilchez R Schulz S Geffers R Yip CY Levesque CM Cvitkovitch DG Wagner-Döbler I Autoinducer-2-regulated genes in Streptococcus mutans UA159 and global metabolic effect of the luxS mutation J Bacteriol 2008 190 1 401 415 10.1128/JB.01086-07 17981981 PMC2223724

[B29] Aharoni R Bronstheyn M Jabbour A Zaks B Srebnik M Steinberg D Oxazaborolidine derivatives inducing autoinducer-2 signal transduction in *Vibrio harveyi* Bioorg Med Chem 2008 16 4 1596 1604 10.1016/j.bmc.2007.11.032 18053731

[B30] Chu F Kearns DB McLoon A Chai Y Kolter R Losick R A novel regulatory protein governing biofilm formation in *Bacillus subtilis* Mol Microbiol 2008 68 5 1117 1127 10.1111/j.1365-2958.2008.06201.x 18430133 PMC2430766

[B31] Kearns DB Division of labour during *Bacillus subtilis *biofilm formation Mol Microbiol 2008 67 2 229 231 18086186 10.1111/j.1365-2958.2007.06053.x

[B32] Bayles KW The biological role of death and lysis in biofilm development Nat Rev Microbiol 2007 5 9 721 726 10.1038/nrmicro1743 17694072

[B33] Kolter R Greenberg EP Microbial sciences: the superficial life of microbes Nature 2006 441 7091 300 302 10.1038/441300a 16710410

[B34] O'Toole GA Stewart PS Biofilms strike back Nat Biotechnol 2005 23 11 1378 1379 10.1038/nbt1105-1378 16273068

[B35] Klein MI Duarte S Xiao J Mitra S Foster TH Koo H Structural and molecular basis of the role of starch and sucrose in Streptococcus mutans biofilm development Appl Environ Microbiol 2009 75 3 837 841 10.1128/AEM.01299-08 19028906 PMC2632160

[B36] Welin J Wilkins JC Beighton D Svensater G Protein expression by *Streptococcus mutans *during initial stage of biofilm formation Appl Environ Microbiol 2004 70 6 3736 3741 10.1128/AEM.70.6.3736-3741.2004 15184181 PMC427790

[B37] Motegi M Takagi Y Yonezawa H Hanada N Terajima J Watanabe H Senpuku H Assessment of genes associated with *Streptococcus mutans *biofilm morphology Appl Environ Microbiol 2006 72 9 6277 6287 10.1128/AEM.00614-06 16957255 PMC1563623

[B38] Wen ZT Baker HV Burne RA Influence of BrpA on critical virulence attributes of *Streptococcus mutans* J Bacteriol 2006 188 8 2983 2992 10.1128/JB.188.8.2983-2992.2006 16585759 PMC1447002

[B39] Harle J Salih V Olsen I Brett P Jones F Tonetti M Gene expression profiling of bone cells on smooth and rough titanium surfaces J Mater Sci Mater Med 2004 15 11 1255 1258 10.1007/s10856-004-5680-1 15880936

[B40] Singh R Paul D Jain RK Biofilms: implications in bioremediation Trends Microbiol 2006 14 9 389 397 10.1016/j.tim.2006.07.001 16857359

[B41] Patel CN Wortham BW Lines JL Fetherston JD Perry RD Oliveira MA Polyamines are essential for the formation of plague biofilm J Bacteriol 2006 188 7 2355 2363 10.1128/JB.188.7.2355-2363.2006 16547021 PMC1428407

[B42] Camilli A Bassler BL Bacterial small-molecule signaling pathways Science 2006 311 5764 1113 1116 10.1126/science.1121357 16497924 PMC2776824

[B43] DeLisa MP Valdes JJ Bentley WE Mapping stress-induced changes in autoinducer AI-2 production in chemostat-cultivated *Escherichia coli *K-12 J Bacteriol 2001 183 9 2918 2928 10.1128/JB.183.9.2918-2928.2001 11292813 PMC99510

[B44] Yuan L Hillman JD Progulske-Fox A Microarray analysis of quorum-sensing-regulated genes in *Porphyromonas gingivalis* Infect Immun 2005 73 7 4146 4154 10.1128/IAI.73.7.4146-4154.2005 15972504 PMC1168601

[B45] Senadheera D Cvitkovitch DG Quorum sensing and biofilm formation by *Streptococcus mutans* Adv Exp Med Biol 2008 631 178 188 full_text 18792689 10.1007/978-0-387-78885-2_12

[B46] Lönn-Stensrud J Petersen FC Benneche T Scheie AA Synthetic bromated furanone inhibits autoinducer-2-mediated communication and biofilm formation in oral streptococci Oral Microbiol Immunol 2007 22 5 340 346 10.1111/j.1399-302X.2007.00367.x 17803632

[B47] Russell MW Harrington DJ Russell RR Identity of *Streptococcus mutans *surface protein antigen III and wall-associated protein antigen A Infect Immun 1995 63 2 733 735 7822052 10.1128/iai.63.2.733-735.1995 PMC173062

[B48] Levesque CM Voronejskaia E Huang YC Mair RW Ellen RP Cvitkovitch DG Involvement of sortase anchoring of cell wall proteins in biofilm formation by *Streptococcus mutans* Infect Immun 2005 73 6 3773 3777 10.1128/IAI.73.6.3773-3777.2005 15908410 PMC1111851

[B49] Qian H Dao ML Inactivation of the *Streptococcus mutans *wall-associated protein A gene (*wapA*) results in a decrease in sucrose-dependent adherence and aggregation Infect Immun 1993 61 12 5021 5028 8225578 10.1128/iai.61.12.5021-5028.1993 PMC281278

[B50] Zhu L Kreth J Cross SE Gimzewski JK Shi W Qi F Functional characterization of cell-wall-associated protein WapA in Streptococcus mutans Microbiology 2006 152 Pt 8 2395 2404 10.1099/mic.0.28883-0 16849803

[B51] Hasona A Crowley PJ Levesque CM Mair RW Cvitkovitch DG Bleiweis AS Brady LJ Streptococcal viability and diminished stress tolerance in mutants lacking the signal recognition particle pathway or YidC2 Proc Natl Acad Sci USA 2005 102 48 17466 17471 10.1073/pnas.0508778102 16293689 PMC1297686

[B52] Keenan RJ Freymann DM Stroud RM Walter P The signal recognition particle Annu Rev Biochem 2001 70 755 775 10.1146/annurev.biochem.70.1.755 11395422

[B53] Herskovits AA Bochkareva ES Bibi E New prospects in studying the bacterial signal recognition particle pathway Mol Microbiol 2000 38 5 927 939 10.1046/j.1365-2958.2000.02198.x 11123669

[B54] Simionato MR Tucker CM Kuboniwa M Lamont G Demuth DR Tribble GD Lamont RJ *Porphyromonas gingivalis *genes involved in community development with *Streptococcus gordonii* Infect Immun 2006 74 11 6419 6428 10.1128/IAI.00639-06 16923784 PMC1695522

[B55] Bustin SA Absolute quantification of mRNA using realtime reverse transcription polymerase chain reaction assays J Mol Endocrinol 2000 25 2 169 193 10.1677/jme.0.0250169 11013345

