# Supplementary material for: Genetic adaptation of Streptococcus mutans during biofilm formation on different types of surfaces
Source: BMC Microbiol. 2010 Feb 18;10:51. doi: 10.1186/1471-2180-10-51 (PMC2838874; doi:10.1186/1471-2180-10-51)
Supplement: Additional file 1 — Figure S1. Schematic diagram showing construction of DNA-microarray experiments for gene expression studies of biofilms on various surfaces. [file 1471-2180-10-51-S1.DOC]

**Composite**

**HA**

**9**

**Ti**

###### Polystyrene

**Figure S1**

Schematic diagram showing construction of DNA-microarray experiments for gene expression studies of biofilms on various surfaces. The sample generated on the polystyrene surface served as a reference for the comparative transcriptome analyses. The arrows indicate the hybridizations performed between the samples generated on specified surfaces.

**Selected genes expression analyzed by RT-PCR**
