# Supplementary material for: Genetic adaptation of Streptococcus mutans during biofilm formation on different types of surfaces
Source: BMC Microbiol. 2010 Feb 18;10:51. doi: 10.1186/1471-2180-10-51 (PMC2838874; doi:10.1186/1471-2180-10-51)
Supplement: Additional file 2 — Table S1. Nucleotide sequences of primers for genes whose expression was compared. Table S2. The differentially expressed (P < 0.05) genes of S. mutans biofilms on HA vs. polystyrene surfaces. Table S3. The differentially expressed (P < 0.05) genes of S. mutans biofilms on composite vs. polystyrene surfaces. Table S4. The differentially expressed (P < 0.05) genes of S. mutans biofilm on Ti vs. polystyrene surfaces. [file 1471-2180-10-51-S2.DOC]

**Table S1.** Nucleotide sequences of primers for genes whose expression was compared

| ORF ^a^ | Primer Sequences (5' – 3') | |
| --- | --- | --- |
|  | Forward | Reverse |
| 16S rRNA | CCTACGGGAGGCAGCAGTAG | CAACAGAGCTTTACGATCCGAAA |
| SMU.81 | AGAAGCACTTGAGCG | ATTAGCGCGACGCTG |
| SMU.82 | GCAGGTCAAGAGGGAGCTCA | CCGCCCTTGTCTGATGAATC |
| SMU.574c | TGGTCATACAGTTGTGCAGC | GACGAGGCCGATGCAACA |
| SMU.609 | CAGTTGTGAACGTGGCTGAAA | TGAGCTGCTGCCTTATCTGAAA |
| SMU.618 | GCACTTATCGCTTGCGGTTT | CACCTGACAATACCAGCAACCA |
| SMU.744 | TGTTCAGGTTGCGTCAACCTT | AATGACACGGCGAAGAGCTT |
| SMU.987 | GCACGCTTGCAGTACATTGC | CATAAGGTCGCGAGCAGCT |
| SMU.1954 | CCAGGAGCTTTGACTGCGAC | TTGCGGATGATGATGTAGATGGTG |

^a^Based on the genome annotation of *S. mutans* provided by TIGR

**Table S2.** The differentially expressed (*P* <0.05) genes of *S. mutans* biofilms on HA vs. polystyrene surfaces*

| **Locus number^a^** | Description^a^ | M^b^ | *P* value^c^ | B^d^ |
| --- | --- | --- | --- | --- |
| **SMU.500** | putative ribosome-associated protein | -3.06081 | 3.05E-11 | 22.19083 |
| **SMU.1602** | putative NAD(P)H-flavin oxidoreductase | -2.00305 | 1.30E-07 | 14.16192 |
| **SMU.1115** | lactate dehydrogenase | -1.41295 | 2.89E-06 | 10.84135 |
| **SMU.1397c** | conserved hypothetical protein | -1.94065 | 1.07E-05 | 9.339835 |
| **SMU.562** | ATP-dependent protease ClpE | -2.17723 | 1.75E-05 | 8.640407 |
| **SMU.1955** | putative co-chaperonin GroES | -1.33543 | 0.00012 | 6.560109 |
| **SMU.1479** | conserved hypothetical protein | -1.04298 | 0.000203 | 5.8948 |
| **SMU.166** | hypothetical protein | -1.07177 | 0.000311 | 5.321531 |
| **SMU.673** | conserved hypothetical protein | 0.963052 | 0.000491 | 4.729395 |
| **SMU.258** | putative oligopeptide ABC transporter, ATP-binding protein OppD | 0.804749 | 0.000491 | 4.600514 |
| **SMU.493** | formate acetyltransferase (pyruvate formate-lyase 2) | -0.86879 | 0.000491 | 4.562527 |
| **SMU.1976c** | hypothetical protein | -0.97795 | 0.000598 | 4.387433 |
| **SMU.81** | heat shock protein GrpE (HSP-70 cofactor) | -0.99735 | 0.000628 | 4.181936 |
| **SMU.598** | putative recombination protein RecM | -0.86033 | 0.000684 | 4.025962 |
| **SMU.1090** | conserved hypothetical protein | -0.7773 | 0.001148 | 3.426349 |
| **SMU.818** | 30S ribosomal protein S21 | -1.20134 | 0.00124 | 3.231515 |
| **SMU.1520** | putative ABC transporter, glutamine binding protein | 0.731843 | 0.00124 | 3.203715 |
| **SMU.618** | hypothetical protein | -1.87625 | 0.00124 | 3.225775 |
| **SMU.495** | glycerol dehydrogenase | -1.12873 | 0.002236 | 2.562159 |
| **SMU.80** | transcriptional regulator repressor (HrcA) of class I | -1.00063 | 0.002236 | 2.514651 |
| **SMU.1910c** | hypothetical protein | 0.939681 | 0.002363 | 2.36614 |
| **SMU.672** | isocitrate dehydrogenase | 1.353307 | 0.002363 | 2.338887 |
| **SMU.1908c** | hypothetical protein | 1.328363 | 0.002363 | 2.323624 |
| **SMU.1745c** | putative transcriptional regulator | 1.201517 | 0.002534 | 2.303972 |
| **SMU.987** | cell wall-associated protein precursor WapA | -1.51424 | 0.002658 | 2.09846 |
| **SMU.1339** | putative bacitracin synthetase | -1.09968 | 0.002658 | 2.066523 |
| **SMU.629** | putative manganese-type superoxide dismutase, Fe/Mn-SOD | -0.76722 | 0.002868 | 1.943892 |
| **SMU.82** | heat shock protein, DnaK (HSP-70) | -0.93303 | 0.002868 | 1.941162 |
| **SMU.2016** | 50S ribosomal protein L24 | 0.91169 | 0.002894 | 1.898196 |
| **SMU.173** | putative ppGpp-regulated growth inhibitor | -0.816 | 0.002923 | 1.833875 |
| **SMU.1954** | putative chaperonin GroEL | -1.63425 | 0.002945 | 1.794535 |
| **SMU.1902c** | hypothetical protein | -1.23047 | 0.00298 | 1.838534 |
| **SMU.1735** | putative acetyl-CoA carboxylase beta subunit | 0.902392 | 0.003011 | 1.711851 |
| **SMU.1975c** | conserved hypothetical protein possible membrane protein | -1.20746 | 0.003108 | 1.680491 |
| **SMU.1388** | putative RNA helicase | -0.89221 | 0.003108 | 1.736587 |
| **SMU.2020** | 50S ribosomal protein L16 | 0.643392 | 0.003175 | 1.595862 |
| **SMU.1705** | hypothetical protein | -0.90049 | 0.003263 | 1.650961 |
| **SMU.299c** | putative bacteriocin peptide precursor | -0.88856 | 0.003263 | 1.516204 |
| **SMU.671** | citrate synthase | 0.867741 | 0.003309 | 1.499763 |
| **SMU.1737** | putative 3-hydroxymyristoyl-(acyl carrier protein) dehydratase | 0.773236 | 0.003462 | 1.448765 |
| **SMU.549** | putative MurG undecaprenyl-PP-MurNAc-pentapeptide-UDPGlcNAc GlcNAc transferase | 0.681862 | 0.003817 | 1.268441 |
| **SMU.251** | conserved hypothetical protein possible ABC transporter, membrane | -0.91199 | 0.004725 | 1.056777 |
| **SMU.595** | putative dihydroorotate dehydrogenase dihydroorotate oxidase | -1.14451 | 0.004813 | 1.317361 |
| **SMU.1603** | putative lactoylglutathione lyase | -1.43417 | 0.004813 | 0.973592 |
| **SMU.1977c** | putative transcriptional regulator | -0.94144 | 0.005543 | 0.927687 |
| **SMU.404c** | hypothetical protein | -1.44613 | 0.00555 | 1.07207 |
| **SMU.1178c** | putative amino acid ABC transporter, ATP-binding protein | 0.658614 | 0.00661 | 0.702296 |
| **SMU.1723c** | conserved hypothetical protein | -0.78249 | 0.00661 | 0.626801 |
| **SMU.1400c** | conserved hypothetical protein | -0.85497 | 0.008313 | 0.41103 |
| **SMU.1182** | mannitol-1-phosphate dehydrogenase | -0.74308 | 0.008313 | 0.454605 |
| **SMU.1043c** | putative phosphotransacetylase | -0.72495 | 0.008458 | 0.299877 |
| **SMU.63c** | conserved hypothetical protein | -0.74395 | 0.009283 | 0.190471 |
| **SMU.168** | putative transcriptional regulator | -1.06944 | 0.009807 | 0.118505 |
| **SMU.2035** | conserved hypothetical protein possible bacteriocin immunity protein | -0.84927 | 0.010653 | 0.081696 |
| **SMU.1081c** | conserved hypothetical protein | 0.911458 | 0.011345 | 0.026109 |
| **SMU.1341c** | putative gramicidin S synthetase | 0.979311 | 0.012006 | -0.1491 |
| **SMU.554** | conserved hypothetical protein | 0.75274 | 0.012079 | -0.05933 |
| **SMU.2105** | hypothetical protein | -1.28185 | 0.012139 | -0.15689 |
| **SMU.1080c** | conserved hypothetical protein possible transposon-related protein | 0.680127 | 0.012193 | -0.17811 |
| **SMU.2011** | 50S ribosomal protein L6 (BL10) | 0.809007 | 0.012571 | -0.24309 |
| **SMU.1340** | putative surfactin synthetase | 0.663507 | 0.012641 | -0.26456 |
| **SMU.1324** | putative cell-division protein FtsX | -1.13554 | 0.012883 | -0.23778 |
| **SMU.1041** | putative ABC transporter, ATP-binding protein | -0.93063 | 0.013265 | -0.28177 |
| **SMU.1562** | putative potassium uptake protein TrkA | -0.98594 | 0.014393 | -0.43693 |
| **SMU.779** | putative 3-dehydroquinate synthase | 0.582647 | 0.015128 | -0.50959 |
| **SMU.1722c** | putative integral membrane protein | -0.76083 | 0.015128 | -0.53173 |
| **SMU.2057c** | putative cadmium-transporting ATPase P-type ATPase | -1.07143 | 0.015501 | -0.56982 |
| **SMU.1958c** | putative PTS system, mannose-specific IIC component | -0.52162 | 0.015682 | -0.59532 |
| **SMU.1360c** | hypothetical protein | 0.760131 | 0.017772 | -0.47612 |
| **SMU.99** | fructose-1,6-biphosphate aldolase | -1.1621 | 0.018951 | -0.7898 |
| **SMU.1005** | glucosyltransferase-SI | 0.894337 | 0.019667 | -0.74393 |
| **SMU.275** | putative L-ribulose 5-phosphate 4-epimerase | -0.66778 | 0.020186 | -0.78261 |
| **SMU.1834** | putative alanine racemase | -0.76726 | 0.021044 | -0.93151 |
| **SMU.202c** | hypothetical protein | -0.81374 | 0.021206 | -0.81716 |
| **SMU.1853** | conserved hypothetical protein | 0.586125 | 0.021206 | -0.92845 |
| **SMU.2066c** | putative transmembrane protein | 0.974717 | 0.021683 | -0.90953 |
| **SMU.167** | hypothetical protein | -0.57766 | 0.021683 | -1.00125 |
| **SMU.1247** | putative enolase | -0.82573 | 0.021683 | -1.0243 |
| **SMU.1678** | conserved hypothetical protein, possible acyl-CoA thioesterase | -0.9954 | 0.023161 | -0.93694 |
| **SMU.1173** | putative O-acetylhomoserine sulfhydrylase | 0.905599 | 0.025581 | -1.22323 |
| **SMU.1922** | putative chromosome replication protein | 0.857318 | 0.026698 | -0.92168 |
| **SMU.1063** | putative ABC transporter, ATP-binding protein, proline/glycine betaine | 0.680317 | 0.026698 | -1.15785 |
| **SMU.1615c** | conserved hypothetical protein | 1.199776 | 0.026785 | -1.19722 |
| **SMU.789** | conserved hypothetical protein | -0.7029 | 0.026785 | -1.18976 |
| **SMU.1706** | conserved hypothetical protein | -0.71984 | 0.026785 | -1.31543 |
| **SMU.2127** | putative succinate semialdehyde dehydrogenase | -0.91443 | 0.026785 | -1.3218 |
| **SMU.427** | putative copper chaperone | -1.13414 | 0.027534 | -1.35943 |
| **SMU.1828** | conserved hypothetical protein | -0.80028 | 0.028802 | -1.42847 |
| **SMU.881** | sucrose phosphorylase, GtfA | 0.861974 | 0.02951 | -1.43969 |
| **SMU.609** | putative 40K cell wall protein precursor | -0.42137 | 0.029525 | -1.45908 |
| **SMU.1029** | conserved hypothetical protein | -1.17388 | 0.029585 | -1.48134 |
| **SMU.1127** | putative 30S ribosomal protein S20 | -0.92081 | 0.029585 | -1.36784 |
| **SMU.2084c** | conserved hypothetical protein | -0.59382 | 0.03023 | -1.52775 |
| **SMU.936** | putative amino acid ABC transporter, ATP-binding protein | -0.93216 | 0.030808 | -1.54163 |
| **SMU.1539** | putative 1,4-alpha-glucan branching enzyme | 0.59411 | 0.034835 | -1.69337 |
| **SMU.744** | putative cell division protein FtsY signal recognition | -1.46747 | 0.034835 | -1.62328 |
| **SMU.494** | putative transaldolase | -0.9235 | 0.035387 | -1.71866 |
| **SMU.151** | hypothetical protein | -0.83092 | 0.036519 | -1.7022 |
| **SMU.1157c** | conserved hypothetical protein | 0.688152 | 0.036519 | -1.45379 |
| **SMU.466** | cysteine aminopeptidase C | -0.81733 | 0.036519 | -1.76384 |
| **SMU.910** | glucosyltransferase-S | -1.04653 | 0.036892 | -1.69626 |
| **SMU.435** | putative N-acetylglucosamine-6-phosphate deacetylase | -0.56178 | 0.036892 | -1.77699 |
| **SMU.574c** | putative membrane protein | -0.4798 | 0.037559 | -1.84472 |
| **SMU.1174** | ATP-dependent DNA helicase | 0.799902 | 0.037559 | -1.83566 |
| **SMU.1536** | putative starch (bacterial glycogen) synthase | 1.320252 | 0.037559 | -1.83737 |
| **SMU.2009** | 30S ribosomal protein S5 | 0.532723 | 0.03988 | -1.91732 |
| **SMU.1018** | hypothetical protein | 0.559259 | 0.039899 | -1.88985 |
| **SMU.781** | putative prephenate dehydrogenase | 0.509913 | 0.039926 | -1.93757 |
| **SMU.1761c** | conserved hypothetical protein | 0.74079 | 0.039926 | -1.87616 |
| **SMU.503c** | hypothetical protein | -0.61424 | 0.04099 | -1.9101 |
| **SMU.2128** | putative dihydroxy-acid dehydratase | -1.12192 | 0.042563 | -2.02321 |
| **SMU.1010** | putative citrate lyase ligase | 1.157006 | 0.043008 | -1.41472 |
| **SMU.1664c** | putative acetoin utilization protein, acetoin dehydrogenase | -1.37236 | 0.04411 | -1.96626 |
| **SMU.880** | multiple sugar-binding ABC transporter, permease protein MsmG | 0.47747 | 0.044684 | -2.08968 |
| **SMU.1611c** | putative permease possible multi-drug resistance efflux pump | 1.356329 | 0.044684 | -1.47462 |
| **SMU.886** | galactokinase, GalK | 0.456667 | 0.048874 | -2.19643 |

^*^ Genes are listed according to their decreasing statistical importance

*^a^* Based on the genome annotation of *S. mutans* provided by TIGR

*^b^* Fold expression change according to the M value (Log_2_Ratio), M>0 means upregulation and M<0 downregulation of the gene

^c^ *P* value, adjusted by Benjamini and Yekutiely method

*^d^* Bayesian test value, that means the probability for a gene to be real differentially expressed.

**Table S3.** The differentially expressed (*P* <0.05) genes of *S. mutans* biofilms on composite vs. polystyrene surfaces*

| **Locus number^a^** | Description^a^ | M^b^ | *P* value^c^ | B^d^ |
| --- | --- | --- | --- | --- |
| **SMU.1424** | putative dihydrolipoamide dehydrogenase | -1.03807 | 0.000102 | 8.305691 |
| **SMU.2105** | hypothetical protein | 2.219075 | 0.000276 | 6.39423 |
| **SMU.1902c** | hypothetical protein | 1.726425 | 0.000276 | 6.372289 |
| **SMU.307** | glucose-6-phosphate isomerase | 2.21823 | 0.0004 | 5.487923 |
| **SMU.1324** | putative cell-division protein FtsX | 1.831063 | 0.0004 | 5.539169 |
| **SMU.598** | putative recombination protein RecM | 0.94188 | 0.000475 | 5.164017 |
| **SMU.1957** | putative PTS system, mannose-specific IID component | 0.883246 | 0.00072 | 4.602723 |
| **SMU.1414c** | conserved hypothetical protein | 1.183637 | 0.000731 | 4.500943 |
| **SMU.2084c** | conserved hypothetical protein | 0.989298 | 0.000896 | 4.154478 |
| **SMU.1022** | conserved hypothetical protein, CitG homolog | -0.9703 | 0.00107 | 3.904695 |
| **SMU.924** | thiol peroxidase | 1.1437 | 0.002161 | 3.160537 |
| **SMU.714** | translation elongation factor EF-Tu | 1.340326 | 0.00225 | 2.985285 |
| **SMU.1900** | conserved hypothetical protein | -1.4088 | 0.00225 | 2.985564 |
| **SMU.340** | 50S ribosomal protein L34 | 1.132318 | 0.00258 | 2.782679 |
| **SMU.2047** | putative PTS system, glucose-specific IIABC component | 1.086304 | 0.002582 | 2.675426 |
| **SMU.359** | translation elongation factor G (EF-G) | 1.761416 | 0.002688 | 2.553232 |
| **SMU.483** | putative phosphoprotein phosphatase (pppL protein) | -0.78732 | 0.003052 | 2.435306 |
| **SMU.318** | putative hippurate hydrolase | 0.777397 | 0.003093 | 2.328027 |
| **SMU.1626** | 50S ribosomal protein L1 | 0.778797 | 0.003721 | 2.077543 |
| **SMU.588** | conserved hypothetical protein | 0.753123 | 0.005215 | 1.727526 |
| **SMU.1255c** | hypothetical protein | 1.303467 | 0.006326 | 1.533654 |
| **SMU.866** | conserved hypothetical protein | 1.497946 | 0.007208 | 1.336691 |
| **SMU.575c** | putative membrane protein | -1.06602 | 0.007208 | 1.224899 |
| **SMU.1809** | putative bacteroiocin operon protein ScnG homolog | -0.66327 | 0.007208 | 1.304535 |
| **SMU.995** | putative ABC transporter, permease protein possible ferrichrome | 0.866763 | 0.00749 | 1.337884 |
| **SMU.1975c** | conserved hypothetical protein possible membrane protein | 1.124382 | 0.008262 | 1.029137 |
| **SMU.865** | 30S ribosomal protein S16 | 1.375979 | 0.008262 | 1.036394 |
| **SMU.1844** | sucrose operon repressor | 0.818244 | 0.008427 | 0.924794 |
| **SMU.258** | putative oligopeptide ABC transporter, ATP-binding protein OppD | -0.58654 | 0.009132 | 0.807997 |
| **SMU.1687** | putative manganese-dependent inorganic pyrophosphatase | 0.950531 | 0.009132 | 0.810501 |
| **SMU.1664c** | putative acetoin utilization protein, acetoin dehydrogenase | 1.717519 | 0.009132 | 0.787025 |
| **SMU.1200** | putative ribosomal protein S1 sequence specific DNA-binding | 1.25244 | 0.009132 | 0.777522 |
| **SMU.618** | hypothetical protein | 1.479121 | 0.009986 | 0.646062 |
| **SMU.987** | cell wall-associated protein precursor WapA | 1.303127 | 0.010269 | 0.550678 |
| **SMU.1948** | putative preprotein translocase subunit SecE | 0.606484 | 0.010842 | 0.527182 |
| **SMU.1412c** | putative ABC transporter, membrane protein subunit and | 0.679626 | 0.013405 | 0.305363 |
| **SMU.1956c** | hypothetical protein | 0.903714 | 0.013405 | 0.207037 |
| **SMU.356** | purine operon repressor | 0.568394 | 0.013405 | 0.19984 |
| **SMU.2118** | putative ABC transporter osmoprotectant-binding protein, glycine betaine/carnitine/choline | 0.662251 | 0.013405 | 0.205098 |
| **SMU.1267c** | hypothetical protein | 0.635057 | 0.013762 | 0.157591 |
| **SMU.1023** | putative pyruvate carboxylase/oxaloacetate decarboxylase, alpha subunit | -0.7369 | 0.013918 | 0.074451 |
| **SMU.80** | transcriptional regulator repressor (HrcA) of class I | 0.79978 | 0.013918 | 0.080978 |
| **SMU.154** | 30S ribosomal protein S15 | 0.741264 | 0.01523 | -0.02722 |
| **SMU.1043c** | putative phosphotransacetylase | 0.694318 | 0.015588 | -0.07124 |
| **SMU.1423** | putative pyruvate dehydrogenase, TPP-dependent E1 component alpha-subunit | -0.65273 | 0.016182 | -0.14975 |
| **SMU.390** | hypothetical protein | 0.93303 | 0.016761 | -0.20412 |
| **SMU.1021** | putative citrate lyase, alfa subunit | -0.96406 | 0.017401 | -0.24557 |
| **SMU.777** | putative 3-dehydroquinate dehydratase | -0.7171 | 0.017401 | -0.24742 |
| **SMU.1046c** | putative GTP pyrophosphokinase | -1.43402 | 0.019371 | 0.067783 |
| **SMU.2119** | putative osmoprotectant ABC transporter permease protein | 0.827747 | 0.020505 | -0.37222 |
| **SMU.1348c** | putative ABC transporter, ATP-binding protein | -0.62487 | 0.020505 | -0.48443 |
| **SMU.963c** | conserved hypothetical protein putative deacetylase | -0.74697 | 0.020505 | -0.51843 |
| **SMU.1163c** | putative ABC transporter, ATP-binding protein | 1.029089 | 0.020505 | -0.44369 |
| **SMU.996** | putative ABC transporter, permease protein possible ferrichrome | 0.939043 | 0.021346 | -0.52238 |
| **SMU.1896c** | hypothetical protein | -0.72798 | 0.021346 | -0.58803 |
| **SMU.744** | putative cell division protein FtsY signal recognition | 1.60433 | 0.021346 | -0.5669 |
| **SMU.1068c** | putative ABC transporter, ATP-binding protein | -0.93057 | 0.021346 | -0.57105 |
| **SMU.935** | putative amino acid ABC transporter, permease protein | 0.534019 | 0.02201 | -0.63673 |
| **SMU.1224** | putative dihydroorotate dehydrogenase, electron transfer subunit | 1.18853 | 0.02201 | -0.3348 |
| **SMU.1467** | putative adenine phosphoribosyltransferase | -0.82133 | 0.02244 | -0.72651 |
| **SMU.1018** | hypothetical protein | -0.63196 | 0.02244 | -0.71933 |
| **SMU.910** | glucosyltransferase-S | 1.075278 | 0.02244 | -0.71476 |
| **SMU.635** | conserved hypothetical protein | -0.66079 | 0.023035 | -0.78452 |
| **SMU.1943** | putative leucyl-tRNA synthetase | 0.747593 | 0.023035 | -0.70748 |
| **SMU.27** | putative acyl carrier protein AcpP; ACP | 0.598535 | 0.023458 | -0.74432 |
| **SMU.2077c** | conserved hypothetical protein | 0.86138 | 0.023527 | -0.75697 |
| **SMU.435** | putative N-acetylglucosamine-6-phosphate deacetylase | 0.632058 | 0.02441 | -0.86923 |
| **SMU.1117** | NADH oxidase (H2O-forming) | -0.66915 | 0.024419 | -0.88775 |
| **SMU.1238c** | conserved hypothetical protein | 0.708375 | 0.024419 | -0.9458 |
| **SMU.427** | putative copper chaperone | 1.156899 | 0.02858 | -1.09922 |
| **SMU.1017** | putative oxaloacetate decarboxylase, sodium ion pump subunit | -0.89376 | 0.02858 | -1.11254 |
| **SMU.1127** | putative 30S ribosomal protein S20 | 0.879481 | 0.02858 | -1.06232 |
| **SMU.594** | hypothetical protein | 0.91789 | 0.031028 | -0.96558 |
| **SMU.78** | fructan hydrolase exo-beta-D-fructosidase; fructanase, FruA | 0.648069 | 0.031028 | -1.21286 |
| **SMU.423** | hypothetical protein | -0.64881 | 0.031028 | -1.17836 |
| **SMU.609** | putative 40K cell wall protein precursor | -0.42749 | 0.031028 | -1.24291 |
| **SMU.1976c** | hypothetical protein | 0.523017 | 0.032413 | -1.27051 |
| **SMU.2008** | 50S ribosomal protein L30 | 0.814983 | 0.033888 | -1.35066 |
| **SMU.1292c** | conserved hypothetical protein | -0.66124 | 0.034949 | -1.37216 |
| **SMU.438c** | putative (R)-2-hydroxyglutaryl-CoA dehydratase activator-related protein | 1.156015 | 0.034949 | -0.96011 |
| **SMU.589** | putative DNA-binding protein | 0.677345 | 0.034949 | -1.43561 |
| **SMU.1609c** | putative membrane protein involved in protein secretion | 0.860574 | 0.037067 | -1.40417 |
| **SMU.840c** | hypothetical protein | 0.492657 | 0.037067 | -1.49133 |
| **SMU.1095** | putative choline ABC transporter, osmoprotectant binding protein | 0.772884 | 0.037067 | -1.49759 |
| **SMU.956** | putative Clp-like ATP-dependent protease, ATP-binding subunit | -1.04872 | 0.037067 | -1.53638 |
| **SMU.1421** | putative dihydrolipoamide acetyltransferase, E2 component | -0.59879 | 0.043129 | -1.68972 |
| **SMU.148** | putative alcohol-acetaldehyde dehydrogenase | -0.63254 | 0.045295 | -1.74658 |
| **SMU.191c** | putative integrase | 1.285455 | 0.046066 | -1.33451 |
| **SMU.1704** | conserved hypothetical protein | -0.7053 | 0.046066 | -1.70511 |
| **SMU.461** | putative amino acid ABC transporter, ATP-binding protein | 0.460429 | 0.046119 | -1.7953 |
| **SMU.1768c** | hypothetical protein | 0.646898 | 0.047568 | -1.81487 |
| **SMU.1933c** | conserved hypothetical protein possible cobalt permease | 0.704206 | 0.047793 | -1.81588 |
| **SMU.969** | dihydropteroate synthase | -0.49139 | 0.0492 | -1.83812 |

^*^ Genes are listed according to their decreasing statistical importance

*^a^* Based on the genome annotation of *S. mutans* provided by TIGR

*^b^* Fold expression change according to the M value (Log_2_Ratio), M>0 means upregulation and M<0 downregulation of the gene

^c^ *P* value, adjusted by Benjamini and Yekutiely method

*^d^* Bayesian test value, that means the probability for a gene to be real differentially expressed.

**Table S4.** The differentially expressed (*P* <0.05) genes of *S. mutans* biofilm on Ti vs. polystyrene surfaces*

| **Locus number^a^** | Description^a^ | M^b^ | P value^c^ | B^d^ |
| --- | --- | --- | --- | --- |
| **SMU.209c** | hypothetical protein | 1.660547 | 1.46E-05 | 10.16132 |
| **SMU.202c** | hypothetical protein | 1.484737 | 9.73E-05 | 7.921404 |
| **SMU.204c** | hypothetical protein | 1.707257 | 0.001238 | 5.104484 |
| **SMU.208c** | putative transposon protein possible DNA segregation ATPase | 1.793005 | 0.001478 | 4.690493 |
| **SMU.2077c** | conserved hypothetical protein | 1.425237 | 0.001478 | 4.456619 |
| **SMU.196c** | putative transfer protein | 1.561519 | 0.001678 | 4.164656 |
| **SMU.2078c** | conserved hypothetical protein | 0.88145 | 0.004671 | 2.901096 |
| **SMU.1957** | putative PTS system, mannose-specific IID component | 0.688087 | 0.004671 | 2.769975 |
| **SMU.205c** | hypothetical protein | 1.660024 | 0.004691 | 2.803853 |
| **SMU.340** | 50S ribosomal protein L34 | 0.975634 | 0.007533 | 2.163844 |
| **SMU.1672** | putative ATP-dependent Clp protease, proteolytic subunit | 0.686554 | 0.010483 | 1.751535 |
| **SMU.191c** | putative integrase | 1.429295 | 0.010662 | 1.942735 |
| **SMU.80** | transcriptional regulator repressor (HrcA) of class I | 0.813585 | 0.011107 | 1.45369 |
| **SMU.198c** | putative conjugative transposon protein | 1.258609 | 0.011107 | 1.340964 |
| **SMU.2084c** | conserved hypothetical protein | 0.699595 | 0.011107 | 1.236346 |
| **SMU.197c** | hypothetical protein | 1.320716 | 0.011107 | 1.390999 |
| **SMU.1948** | putative preprotein translocase subunit SecE | 0.592005 | 0.011107 | 1.231194 |
| **SMU.245** | putative negative regulator of genetic competence MecA | 0.532371 | 0.011107 | 1.168972 |
| **SMU.2147c** | conserved hypothetical protein | 0.443544 | 0.011501 | 1.039704 |
| **SMU.200c** | hypothetical protein | 1.344223 | 0.011501 | 1.135501 |
| **SMU.207c** | putative transposon protein | 1.484302 | 0.011501 | 1.11246 |
| **SMU.211c** | hypothetical protein | 1.197101 | 0.013291 | 0.874325 |
| **SMU.2083c** | hypothetical protein | 1.029424 | 0.013291 | 0.797189 |
| **SMU.1763c** | conserved hypothetical protein | 0.830426 | 0.013291 | 0.686837 |
| **SMU.194c** | conserved hypothetical protein Bacteriophage P2 associated | 1.109386 | 0.013291 | 0.738687 |
| **SMU.1902c** | hypothetical protein | 0.959053 | 0.013291 | 0.684663 |
| **SMU.201c** | putative transposon protein | 1.568401 | 0.014255 | 0.666952 |
| **SMU.154** | 30S ribosomal protein S15 | 0.68743 | 0.016142 | 0.351652 |
| **SMU.199c** | hypothetical protein | 1.351183 | 0.016142 | 0.504213 |
| **SMU.2138** | putative replicative DNA helicase (DNA polymerase III | 0.715442 | 0.016272 | 0.439732 |
| **SMU.1761c** | conserved hypothetical protein | 0.821598 | 0.01726 | 0.261931 |
| **SMU.1922** | putative chromosome replication protein | 0.620198 | 0.020142 | 0.142618 |
| **SMU.210c** | hypothetical protein | 1.267491 | 0.020142 | -0.02492 |
| **SMU.1200** | putative ribosomal protein S1 sequence specific DNA-binding | 1.055999 | 0.020142 | 0.018199 |
| **SMU.1010** | putative citrate lyase ligase | 1.070833 | 0.020142 | 0.390426 |
| **SMU.2079c** | conserved hypothetical protein | 1.206979 | 0.022091 | -0.12059 |
| **SMU.1529** | FoF1 membrane-bound proton-translocating ATPase, gamma subunit | -0.79406 | 0.024011 | -0.28564 |
| **SMU.232** | acetolactate synthase, small subunit | -0.5246 | 0.030136 | -0.55011 |
| **SMU.1626** | 50S ribosomal protein L1 | 0.544791 | 0.030136 | -0.55368 |
| **SMU.671** | citrate synthase | -0.62999 | 0.030366 | -0.54994 |
| **SMU.1745c** | putative transcriptional regulator | 0.752933 | 0.032378 | -0.60826 |
| **SMU.447** | conserved hypothetical protein | -0.44729 | 0.032451 | -0.63361 |
| **SMU.1011** | putative CitG protein | 1.004944 | 0.034033 | -0.28967 |
| **SMU.1956c** | hypothetical protein | 0.717127 | 0.042152 | -0.9733 |

^*^ Genes are listed according to their decreasing statistical importance

*^a^* Based on the genome annotation of *S. mutans* provided by TIGR

*^b^* Fold expression change according to the M value (Log_2_Ratio), M>0 means upregulation and M<0 downregulation of the gene

^c^ *P* value, adjusted by Benjamini and Yekutiely method

*^d^* Bayesian test value, that means the probability for a gene to be real differentially expressed.
